# Seed Biopriming With *Trichoderma* Strains Isolated From Tree Bark Improves Plant Growth, Antioxidative Defense System in Rice and Enhance Straw Degradation Capacity

**DOI:** 10.3389/fmicb.2021.633881

**Published:** 2021-02-26

**Authors:** Harekrushna Swain, Totan Adak, Arup K. Mukherjee, Sarmistha Sarangi, Pankajini Samal, Ansuman Khandual, Rupalin Jena, Pratap Bhattacharyya, Soumendra K. Naik, Sayaji T. Mehetre, Mathew S. Baite, Sunil Kumar M, Najam Waris Zaidi

**Affiliations:** ^1^Crop Protection Division, ICAR-National Rice Research Institute, Cuttack, India; ^2^Department of Botany and Biotechnology, Ravenshaw University, Cuttack, India; ^3^Crop Improvement Division, ICAR-National Rice Research Institute, Cuttack, India; ^4^Division of Crop Production, ICAR-National Rice Research Institute, Cuttack, India; ^5^Nuclear Agriculture and Biotechnology Division, Bhabha Atomic Research Centre, Trombay, India; ^6^International Rice Research Institute, New Delhi, India

**Keywords:** *Trichoderma hebeiensis*, indole acetic acid, prussic acid, straw degrading enzyme, vigor index, stress responsive enzyme, antioxidant genes, biofertilizer

## Abstract

This study is a unique report of the utilization of *Trichoderma* strains collected from even tree barks for rice plant growth, its health management, and paddy straw degradation. Seven different spp. of *Trichoderma* were characterized according to morphological and molecular tools. Two of the isolated strains, namely *Trichoderma hebeiensis* and *Trichoderma erinaceum*, outperformed the other strains. Both of the strains controlled four important rice pathogens, i.e., *Rhizoctonia solani* (100%), *Sclerotium oryzae* (84.17%), *Sclerotium rolfsii* (66.67%), and *Sclerotium delphinii* (76.25%). Seed bio-priming with respective *Trichoderma* strains reduced the mean germination time, enhanced the seedling vigor and total chlorophyll content which could be related to the higher yield observed in two rice varieties; Annapurna and Satabdi. All the seven strains accelerated the decomposition of rice straw by producing higher straw degrading enzymes like total cellulase (0.97–2.59 IU/mL), endoglucanase (0.53–0.75 IU/mL), xylanase (145.35–201.35 nkat/mL), and laccase (2.48–12.60 IU/mL). They also produced higher quantities of indole acetic acid (19.19–46.28 μg/mL), soluble phosphate (297.49–435.42 μg/mL), and prussic acid (0.01–0.37 μg/mL) which are responsible for plant growth promotion and the inhibition of rice pathogen populations. Higher expression of defense enzymes like catalase (≥250% both in shoot and root), peroxidase (≥150% in root and ≥100% in shoot), superoxide dismutase (≥ 150% in root and ≥100% in shoot), polyphenol oxidase (≥160% in shoot and ≥120% in shoot), and total phenolics (≥200% in root and ≥250% in shoot) as compared to the control indicates stress tolerance ability to rice crop. The expression of the aforementioned enzymes were confirmed by the expression of corresponding defense genes like PAL (>3-fold), DEFENSIN (>1-fold), POX (>1.5-fold), LOX (>1-fold), and PR-3 (>2-fold) as compared to the non-treated control plants. This investigation demonstrates that *Trichoderma* strains obtained from tree bark could be considered to be utilized for the sustainable health management of rice crop.

## Introduction

The population of the world is increasing rapidly and it is expected that the world population will be around 9.6 billion in 2050. To attain food security for all, the production of food must be increased to 70% by 2050. Crops should be protected from biotic stresses in order to achieve this goal. This should be done in a more eco-friendly and sustainable way, potentially by using certain biocontrol agents (BCA).

Different BCA like bacteria, fungi, and viruses are being used frequently for the management of diseases in different crops (Abraham et al., 2013). Fungal biocontrol agents are popular as they may be reproduced easily in an artificial nutrient media and are suitable for commercial multiplication (Singh et al., 2013). Genus *Trichoderma* is compelling as a biocontrol operator against various pathogens (Parmar et al., 2015). The primary natural habitat of *Trichoderma* is traditionally seen as soil or plant rhizosphere, even though maximum diversity of these species happens over-the-ground (Druzhinina et al., 2011). With the expanding dangers to nature and to our food security, determination of *Trichoderma* spp. as a BCA has been expecting centrality in giving security for plant protection and development (Swain and Mukherjee, 2020). *Trichoderma* spp. also induces plant growth by the creation of various phytohormones and activates plant supplements for better boost. *Trichoderma* spp. is not only marketed as a biopesticide, biofertilizer, and growth promoter, but also used as a nutrient solubilizer and organic matter decomposer (Woo et al., 2014). There are just a couple of reports on the assessment of *Trichoderma* as a biocontrol specialist obtained from above the ground territories (Jahagirdar et al., 2019). As per Whipps and Lumsden (2001), almost 60% of fungal BCA market is shared by *Trichoderma* spp. and there are significant challenges to investigate. The activity or mode of action of *Trichoderma* spp. is as per the following:

1. Generation of trichodermin, trichothecenes, trichorzianins, or gliotoxins (Mukherjee et al., 2013).
2. Seeking sustenance and space (Celar, 2003).
3. Antibiosis (Swain and Mukherjee, 2020).
4. Mycoparasitic capacities—a relationship in which one living fungus goes about as a supplement hotspot for another (Punja and Utkhede, 2003).

*Trichoderma* spp. produces auxins that are chargeable for plant bloom and root improvement in each symbiotic and pathogenic communication with plants (Swain et al., 2018). An amazing effect on plant improvement has been demonstrated for several *Trichoderma* secondary metabolites. Koninginins, 6-pentyl-α-pyrone, trichocaranes A–D, harzianopyridone, cyclonerodiol, harzianolide, and harzianic acid are instances of exacerbates that affect plant development in a considerably subordinate way (Swain and Mukherjee, 2020). To understand the plant growth promotion activity, rice seeds were bioprimed with the *Trichoderma* strains. Biopriming will help in increase in colonization, proliferation, and establishment of BCA on the seed surface. Consequently, it will boost seedling vigor and will be able to induce systemic resistance to biotic and abiotic stresses (Singh D. P. et al., 2020). Plant-microbe interactions setup by distant, distinct, and assorted microbial associations tend to instigate various common beneficial systemic changes in the expression level of plant genes that encode for proteins to detoxify reactive oxygen species (ROS). The beneficial microorganisms because of their presence in the plant rhizosphere help plants in easing biotic and abiotic stresses (Singh P. et al., 2020).

Faster decomposition of rice straw can be achieved by inoculating microorganisms, like ligno-cellulolytic microbes. *Trichoderma* produces high levels of several biomass degrading enzymes like cellulase and xylanase (Juturu and Wu, 2014). These enzymes degrade cellulose and hemicellulose, respectively. Lignin was degraded by ligninolytic enzymes into simpler phenyl rings (Sancez, 2009). In this investigation, we analyzed seven distinctive spp. of *Trichoderm*a segregated from the bark of various trees in the Odisha province of India to consider biocontrol properties and rice straw decomposition capacity alongside their growth promotion activity in rice by the production of various enzymes and the expression of genes related to this.

## Materials and Methods

### Isolation, Characterization, Growth Condition and Biocontrol Potential of *Trichoderma* spp.

Seven *Trichoderma* strains were collected from the bark of various trees. The isolation and purification of all the strains were made according to standard techniques described by Mukherjee et al. (2014). *Trichoderma* species were identified by using the typical conidiophores structure (Gams and Bissett, 1998) and according to ISTH guidelines. Molecular characterization, identification, and construction of the phylogenetic tree of all the fungal isolates were done according to Swain et al. (2018). Total genomic DNA from the young mycelia was secluded by utilizing standard SDS (sodium dodecyl sulfate) technique (Mukherjee et al., 2014). The molecular characterization of all the fungal segregates were based on the sequences of Internal Transcribed Spacer (ITS) regions, Translation Elongation Factor 1 (TEF1) regions and RNA Polymerase B-larger subunit-II (RPB-II) regions according to standard strategies^1^. The species were recognized by BLASTN search on the NCBI site and the character was affirmed by contrasting the sequences and with authentic sequences from GenBank, and a phylogenetic tree constructed on http://www.phylogeny.fr. Biocontrol potential of isolated *Trichoderma* strains was evaluated against above *Rhizoctonia solani* CRRI-RS-8 (MTCC-12232) causing sheath blight of rice, *Sclerotium oryzae* CRRI-S.O (MTCC-12230) causing seedling blight of rice, *Sclerotium rolfsii* causing foot rot of rice and *Sclerotium delphinii* (MTCC11568) causing seedling rot of rice.

The confrontation assay was carried out by concurrent inoculation of both *Trichoderma* and the pathogen close to the edge of the plate, put opposite to one another. Plates inoculated with pathogens only were utilized as control. The percentage of mycelial growth inhibition was determined by Swain et al. (2018)

Percentageofinhibition=[(R1-R2)/R1]×100

where, R1, radial growth of the pathogen in control plate; R2, radial growth of the pathogen in test plate.

### Quantification of Production of Indole Acetic Acid, Prussic Acid, and Solubilization of Inorganic Phosphate by *Trichoderma* spp.

The indole acetic acid (IAA) produced by different strains of *Trichoderma* was quantified as per Sureshrao et al. (2016). For the quantitative estimation of IAA, agar plugs (5 mm) from the edge of actively growing colonies of *Trichoderma* were inoculated to 20 ml DF (Dworkin and Foster) salts minimal media and incubated for 3 days at 28°C. The medium was supplemented with L-tryptophan at a concentration of 1.02 g l^–1^. After incubation for 72 h, the mycelia were removed from the culture medium by centrifugation at 5,000 rpm for 5 min. One ml aliquot of the supernatant was mixed vigorously with 4 ml of Salkowski’s reagent and allowed to stand at room temperature for 20 min. The absorbance at 535 nm was measured. The concentration of IAA in each culture supernatant was determined by using an IAA (Himedia) as standard curve.

For prussic acid production, *Trichoderma* spp. was grown on Tryptic Soya Agar (TSA) supplemented with 4.4 g L^–1^ of glycine for 2 days. White filter paper discs were cut in the same size and soaked in picric acid solution. The sheets of filter papers were placed on the upper lid of each plate. The plates were sealed with Parafilm and incubated for 7 days at 28°C. After incubation, prussic acid production was observed by the color changes of the filter paper from yellow to light brown or reddish brown (Meera and Balabaskar, 2012). The colored filter paper was then eluted by placing the filter paper in a clean test tube containing 10 mL distilled water and the absorbance was measured at 625 nm by using a spectrophotometer (Manwar et al., 2011).

Quantitative estimation of phosphate solubilization was performed in Pikovskaya broth (Himedia) containing tricalcium phosphate as a phosphate source. Freshly grown *Trichoderma* isolates were inoculated to 50 ml of Pikovskaya’s broth and incubated at 28°C and allowed to shake at 100 rpm. After 5 days the broth culture was centrifuged at 10,000 rpm for 10 min. To the 0.5 ml of the culture supernatant, 5 ml of chloromolybdic acid was added and mixed thoroughly. Volume was made up to 10 ml with distilled water and 125 μl chlorostannous acid was added to it. Immediately, the final volume was made-up to 25 ml with distilled water and mixed thoroughly. The absorbance was measured at 610 nm by using a spectrophotometer. The corresponding amount of soluble phosphorous was calculated from a standard curve of potassium dihydrogen phosphate (KH_2_PO_4_). Phosphate solubilizing activity was expressed in terms of tricalcium phosphate solubilization which in turn was measured by μg ml^–1^ of available orthophosphate as calibrated from the standard curve of KH_2_PO_4_ (Jackson, 1973).

### Qualitative and Quantitative Screening of Straw Degrading Enzymes Produced by *Trichoderma* spp.

Seven *Trichoderma* strains were screened for cellulase activity on carboxy-methyl cellulose (CMC) (Analytical Reagent grade, Himedia, India) and xylanase activity on xylan agar medium containing 1% beech wood xylan (Molecular biology grade, Himedia, India) as the substrate (Teather and Wood, 1982). The *Trichoderma* isolates were cultured on petriplates (90 mm × 15 mm, Himedia, India) containing 1% CMC agar media or 1% beech wood xylan media at 26°C. After 5 days’ incubation, plates were stained with 1% solution of Congo red and followed by destained with 1N NaOH solution on a Gyrotory Shaker (Model G2, New Brunswick Scientific Co., Inc., Edison, NJ, United States) at 50 rpm for 15 min to detect clear distinct zone (Saczi et al., 1986). The Enzymatic Index (EI) was calculated for the above two enzymes by measuring the clearance zone as per Florencio et al. (2012).

The qualitative screening for laccase activity of the *Trichoderma* strains were examined by culturing the respective isolates on PDA media supplemented with 0.04% guaiacol (Extra Pure, Himedia, India) and 0.01% (w/v) chloramphenicol with pH 5.5. They were examined for the development of a mixture of red-brown colored zone around the fungal colonies after incubation at 28°C for 72 h (Kalra et al., 2013). Here; three independent experiments were performed with three imitates per isolate. For each isolate the average EI of the three analyses was determined alongside the standard deviation.

The quantitative enzymatic screening of seven *Trichoderma* strains was carried out. Assay of four major enzymes, i.e., total cellulase, endoglucanase, xylanase, and laccase were carried out. The extraction of the crude enzyme was done using rice straw (Variety, Swarna sub-1, *Indica type*) as the base material as per Pathak et al. (2014) with minor modifications. *Trichoderma* grown on PDA plates (2 days’ culture) were sub-cultured and grown on rice bran (4%) agar media for a period of 5 days at 27°C. After completion of the incubated period, 10 mL of sterile double distilled water having 0.1% polyoxyethylene (20) sorbitan monooleate was added to the plates to collect the fungal spores. Fungal spores (2 × 10^6^ CFU/mL) were inoculated in 250 mL flask containing grinded sterilized rice straw of 5 g and 15 mL of Mandel and Reese nutrient salt solution (NSS). After 5 days of incubation, crude enzyme extraction was done by using citrate buffer (pH 4.8) in NSS: extraction buffer (1:2: V/V). The fermented matter was shaken for 15 min at room temperature. Multilayered cheese cloth was used for the filtration of the fermented product and the centrifugation of the filtrate was done at 10,000 rpm for 15 min at 4°C (Hermel, Labortechnik GmbH, Type-Z36HK, Nr-580901000). The clear supernatant was used as a crude enzyme sample.

The total cellulase activity and endo-β-1,4-glucanase of the above *Trichoderma* strains was determined as units per milliliter (IU/mL) by Dinitro Salicylic Acid (DNS) method using Whatman No.1 filter paper and 2% CMC as substrates, respectively (Ghosh, 1987). The activity of xylanase and laccase enzyme produced by the *Trichoderma* isolates were measured by using 1% xylan (Bailey et al., 1992) and guaiacol (Monssef et al., 2016) as substrates, respectively. The activity of xylanase and laccase was expressed in nkat/mL and IU/mL, respectively.

### *In vitro* Preparation of Rice Straw Compost by *Trichoderma* spp.

The *in vitro* preparation of rice straw compost was carried out by all the strains of *Trichoderma*. The moisture content in rice straw was measured. Inoculums of seven different isolates (10^7^ cfu mL^–1^) were poured into different conical flasks (250 mL) containing 25 gm of straw (Variety, Swarna sub-1, *Indica type*) and kept at ambient temperature. The weight of the flask was measured at regular intervals along with control until 60 days after inoculation. The C/N ration of the decomposed rice straw was determined. Total carbon and nitrogen content was measured according to the methods given by Walkley and Black (1934) and Keeney and Bremner (1965), respectively.

### Measurement of Plant Growth Promoting Parameters, Vigor Index, and Chlorophyll Content Under Greenhouse Conditions

Biocontrol potential and growth promotion property of *Trichoderma* strains were confirmed under controlled conditions. Rice seeds (Annapurna, *Indica* type and Satabdi, *Indica* type) were bio-primed with respective *Trichoderma* formulations (1 × 10^7^ cfu g^–1^) and set in soils (double autoclaved) filled in 30 cm × 30 cm pots. Soapstone (a mix of Talcum powder, hydrous magnesium silicate, and Talc) was used to prepare the respective *Trichoderma* formulation as per Indian Patent File no. 1240/KOL/2015. Seeds not primed with *Trichoderma* formulation were treated as control. Germination capacity and vigor index of the seeds sown in the pot were examined as per Abdul-Baki and Anderson (1973) and Swain et al. (2018). For the assessment of vigor index germination percentage, seedling length and dry weight of seedling was taken into consideration. The effect of *Trichoderma* on the plant chlorophyll content of the leaf was evaluated as per Porra (2002).

### Measurement of Plant Growth Promoting Parameters Under Field Conditions

For the assessment of growth promotion under *in vivo* conditions two direct seeded rice varieties (“Annapurna” and “Satabdi”) were used. Seeds dressed with respective *Trichoderma* formulations were treated as treatments. The experiment was conducted in completely randomized design with four replications. The agronomical parameters (root length, shoot length, dry root weight, dry shoot weight, no of tiller/hill, and yield/hill) were recorded. Expression of plant stress responsive enzymes like peroxidase (PER), catalase (CAT), superoxide dismutase (SOD), polyphenol oxidase (PPO), and total phenolics (TP) conveyed by the plants treated with *Trichoderma* were investigated at active tillering stage (Arnon, 1949; Sadasivam and Manickum, 2011; Swain et al., 2018).

### Extraction of RNA, cDNA Synthesis and Examination of Gene Articulation by Real-Time-PCR

Total RNA was extracted from the fresh plant leaves by utilizing RNeasy Plant Mini Kit (Qiagen, Germany). RNA concentration was quantified by using Nano Drop 2000 (Thermo Fisher Scientific). cDNA synthesis was carried out by using Maxima H minus first strand cDNA synthesis Kit with dsDNase (Thermo Fisher Scientific) as per the manufacturer’s guidelines. Quantitative real-time (RT-PCR) reaction was performed in the Insta-Q96 RT-PCR system (Himedia Laboratories, Mumbai, India) using Maxima SYBRGreen/ROX qPCR master mix (Thermo Fisher Scientific) for five growth promotion and antioxidant genes, i.e., POX (peroxidase), LOX (lipoxigenase), PAL (phenylalanine ammonia lyase), DEFENSIN, PR-3 (PR protein). Act (actin) was utilized as housekeeping genes for normalization of relative gene articulation level. All primers were designed by IDT programming. All the treatments were in sets of three for each primer pair in a similar plate. The PCR reactions were set by convention formulated by Singh P. et al. (2020) with minor adjustments.

### Statistical Analysis

Statistical analysis was performed by utilizing the Statistical Analysis Software (SAS) of ICAR-IASRI, New Delhi^2^ was used for statistical analysis. All the investigations were replicated three times. Seed germination rate was ARCSINE transformed. The other seed quality boundaries were examined with no change. All the information was exposed to single way characterized analysis of variance (ANOVA) and means of treatments were compared based on Tukey’s honestly significant difference test (HSD) at 0.05 probability level using SAS.

## Results and Discussion

### Identification of *Trichoderma* Strains

**FIGURE 1:**
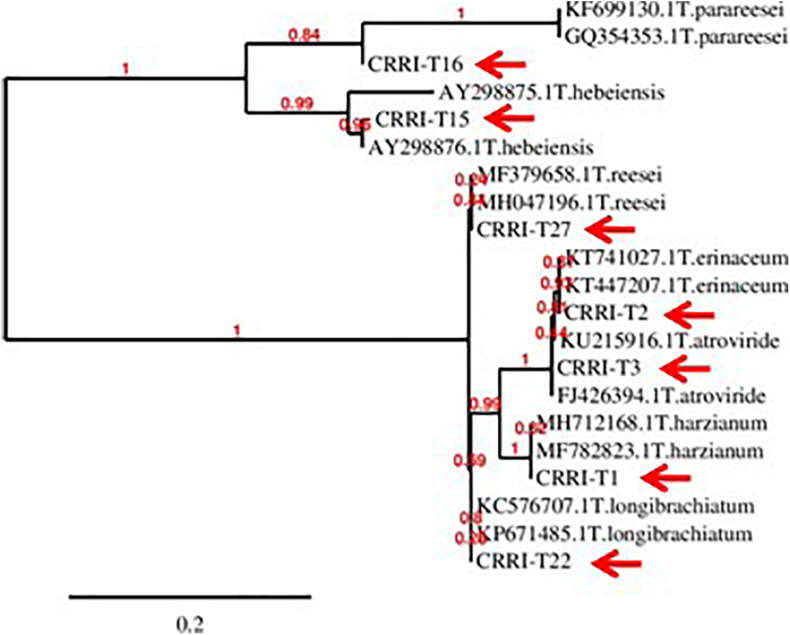
Phylogeny of the isolated *Trichoderma* spp. (CRRI-T1 to CRRI-T27) used for the recent study.

Based on morphological characteristics and molecular identification, the isolates were identified as *Trichoderma harzianum* (CRRI-T1), *Trichoderma erinaceum* (CRRI-T2), *Trichoderma atroviride* (CRRI-T3), *Trichoderma hebeiensis* (CRRI-T15), *Trichoderma parareesei* (CRRI-T16), *Trichoderma longibrachiatum* (CRRI-T22), and *Trichoderma reesei* (CRRI-T27) (Figure 1 and Supplementary Figure 1). The ITS sequence data from CRRI-T1, CRRI-T2, CRRI-T3, CRRI-T22, CRRI-T27, and TEF sequence data from CRRI-T15, CRRI-T16 have been deposited with NCBI (Table 1). Out of these 7 isolates 4 are rarely found in India (i.e., *T. erinaceum, T. hebeiensis, T. parareesei*, and *T. reesei*). This provides the evidence for the maximum diversity of this genus occurring above ground. This may be the first time in India, we are reporting *T. hebeiensis, T. parareesei*, and *T. reesei* from the above ground. *T. reesei* reported earlier were isolated from soil and obtained from the mutation of other species of *Trichoderma* (Kar et al., 2006; Saravanan et al., 2008).

**TABLE 1:** Details of *Trichoderma* isolates and confrontation assay showing the inhibition of pathogen growth by different *Trichoderma* isolates on PDA medium.

Strain designation	Source of collection	Place of collection	GPS location	Species identified	NCBI accession Nos.	Percentage of inhibition			
						*Rhizoctonia solani*	*Sclerotium oryzae*	*Sclerotium rolfsii*	*Sclerotium delphinii*
CRRI-T1	Bark of a *Litchi chinensis*	NRRI, Cuttack	85°92′E, 20°45′N	*Trichoderma harzianum*	KX853519.1	98.75^A^	67.50^C^	40.83^D^	63.33^B^
CRRI-T2	Bark of a *Cassia tora*	42-Mouza (Barala), Cuttack	86°92′E, 20°44′N	*Trichoderma erinaceum*	KR014407.1	100.00^A^	73.33^B^	49.58^C^	75.00^A^
CRRI-T3	Bark of a *Cassia tora*	42-Mouza (Barala), Cuttack	86°58′E, 20°45′N	**Trichoderma atroviride**	KR014408.1	100.00^A^	70.83^BC^	47.50^C^	56.67^C^
CRRI-T15	Bark of a *Samanea saman*	42-Mouza (Barala), Cuttack	86°52′E, 20°45′N	*Trichoderma hebeiensis*	MK247223.1	100.00^A^	84.17^A^	66.67^A^	76.25^A^
CRRI-T16	Bark of a *Samanea saman*	42-Mouza (Barala), Cuttack	86°12′E, 20°44′N	*Trichoderma parareesei*	MK247224.1	100.00^A^	69.58^BC^	57.50^B^	58.75^BC^
CRRI-T22	Decomposed wood of *Dalbergia sissoo*	NRRI, Cuttack	85°92′E, 20°45′N	*Trichoderma longibrachiatum*	MH894348.1	98.33^A^	18.75^E^	14.17^E^	59.58^BC^
CRRI-T27	Bark of a *Samanea saman*	Fakirpada, Cuttack	86°92′E, 20°45′N	*Trichoderma reesei*	MK163352.1	99.58^A^	31.67^D^	45.42^CD^	59.17^BC^
					CV (%)	0.65	3.05	5.01	1.585
					Tukey’s HSD at 5%	1.855	5.1802	6.5756	5.5483

*Means with same letter are not significantly different at p ≤ 0.05.*

### Biocontrol Capability of *Trichoderma* Strains and Its Mechanism

Mycelial growth of *R. solani, S. oryzae, S. rolfsii*, and *S. delphinii* were inhibited by 98.33–100.00, 18.75–84.17, 14.17–66.67, and 56.67–76.25%, respectively. CRRIT-2, CRRIT-3, CRRIT-15, CRRIT-16 overgrew *R. solani* within 3 days. Similarly, these isolates grew quicker in dual culture against *S. oryzae* and covered minimum of 70% of the medium surface within 3 days (Table 1). In case of *S. delphinii*, CRRIT-15, CRRIT-2 exhibited more than 70% inhibitory effect whereas all other strains were able to colonize in between 58 and 63% of the medium surface. Among the seven strains, *T. hebeiensis* (CRRIT-15) and *T. erinaceum* (CRRIT-2) was superior antagonist against four rice pathogens (Supplementary Figure 2). Biocontrol potential of these isolates can be correlated with prussic acid (HCN) production. The highest quantity of HCN was produced by CRRI-T15 (0.37 μg/mL) which was significantly higher as compared to other isolates. Other isolates such as CRRI-T1, CRRI-T2, CRRI-T3, CRRI-T16, and CRRI-T27 produced 0.03, 0.02, 0.05, 0.02, and 0.03 μg/mL HCN (Table 2).

**TABLE 2:** Quantitative enzyme assay of selected *Trichoderma* isolates.

Treatment name	IAA (in μ g/mL)	HCN (in μ g/mL)	Inorganic phosphate (in μ g/mL)	Endoglucanase (in IU/mL)	Total cellulase (in IU/mL)	Xylanase (in nkat/mL)	Laccase (in IU/mL)
CRRIT-1	31.37^C^	0.03^C^	318.20^D^	0.67^C^	0.99^E^	145.35^E^	2.64^C^
CRRIT-2	42.38^B^	0.27^B^	412.30^B^	0.70^BC^	0.87^F^	195.89^B^	11.74^A^
CRRIT-3	30.39^C^	0.05^C^	339.30^C^	0.68^C^	2.13^B^	192.99^B^	9.85^B^
CRRIT-15	46.28^A^	0.37^A^	435.42^A^	0.75^A^	2.59^A^	201.35^A^	12.60^A^
CRRIT-16	22.42^D^	0.02^C^	303.70^DE^	0.59^D^	1.09^D^	152.43^D^	3.35^C^
CRRIT-22	19.19^E^	0.01^C^	297.49^E^	0.53^E^	0.97^E^	149.29^DE^	2.48^C^
CRRIT-27	32.01^C^	0.03^C^	320.20^D^	0.74^AB^	1.28^C^	164.40^C^	2.87^C^
CV (%)	3.48	18.93	1.74	2.38	1.64	0.85	6.99
Tukey’s HSD at 5%	3.1823	0.0601	17.2	0.0453	0.0665	4.1534	1.2982

*Means with same letter are not significantly different at p ≤ 0.05.*

Many species of *Trichoderma* were accounted for as biocontrol specialists for a wide range of plant pathogens (Abdollahi et al., 2012; Kumar et al., 2012; Swain et al., 2018). Mycoparasitism is clearly one of the mechanisms for biocontrol action of *Trichoderma* (Mukherjee et al., 2013). Besides mycoparasitism, release of prussic acid has been proposed as a significant antifungal component. Cyanide produced by microbes may act as an inhibitor to soil borne pathogens without any harm to the host plant (Noori and Saud, 2012). Hence, for the management of soil borne pathogens, the prussic acid produced by *Trichoderma* spp. played a vital role. Biocontrol potential of *Trichoderma* spp. mainly depends on the host plant, agro climatic conditions and nutrient availability (Mukherjee et al., 2013). To the best of our knowledge, *T. hebeiensis* and *T. pararees*ei from the above ground part have been explored as potential biocontrol for the first time.

### *Trichoderma* as a Decomposer

**FIGURE 2:**
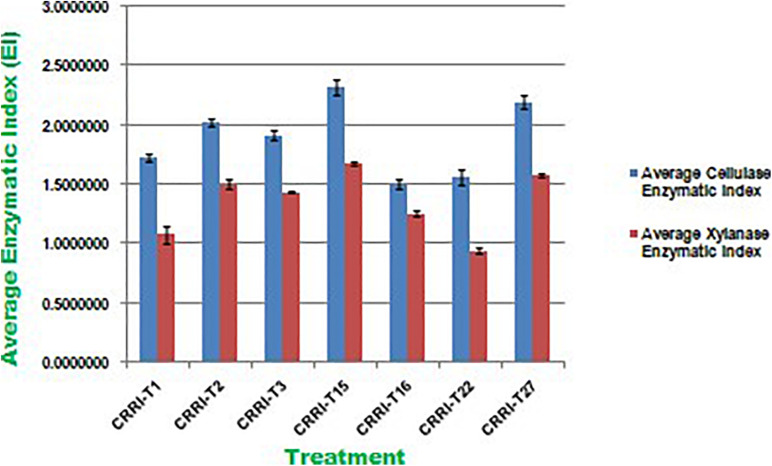
Enzymatic Index of cellulase and xylanase activity of Trichoderma isolates used in the present study.

Cellulase activity of fungal isolates with EI values of more than 1.5 were considered to be potential cellulase producers (Florencio et al., 2012; Saroj et al., 2018). All the seven strains were potential cellulase producers as the EI value was more than 1.5. Earlier Saroj et al. (2018) reported thermophilic fungi isolated from soil, i.e., *Aspergillus fumigatus JCM 10253* and *Aspergillus terreus* with highest EI values of 1.50 and 1.24 for cellulase activity, respectively. Florencio et al. (2012) reported *T. harzianum* CEN 139, *T.* sp. 104 NH and *T. harzianum* CEN 155 exhibited 1.74, 1.72, and 1.61 EI value for cellulase activity, respectively. Similarly, EI values of xylanase activity of *Trichoderma* isolates ranged from 0.50 to 1.33. CRRIT-15 exhibited the highest xylanase activity, i.e., 1.68 EI value followed by CRRIT-27 and CRRIT-2. The strains reported by Saroj et al. (2018) exhibited range 1.01–1.13 of EI value for xylanase activity. However, CRRIT-26, CRRIT-2, and CRRIT-27 had better cellulase and xylanase activities in comparison to others as reported earlier. All the strains exhibited positive reddish-brown zones around the fungal colonies indicating laccase activity. Monssef et al. (2016) examined 24 fungal isolates for the production laccase enzyme and found only *T. harzianum* could produce the enzyme. These results are in line with Gochev and Krastanov (2007) who found that many of *Trichoderma* spp. with cellulase activity could also be a good source of laccase (Figure 2).

All the isolates were examined for endoglucanase, total cellulase, xylanase, and laccase activity. CRRIT-15 showed maximum endoglucanase activities, i.e., 0.75 IU/mL whereas CRRIT-22 isolate showed lowest endoglucanase activities (i.e., 0.53 IU/mL). Similarly, CRRIT-15 and CRRIT-3 showed maximum total cellulase activities, i.e., 2.59 and 2.13 IU/mL, respectively. Similarly, CRRIT-15 released maximum xylanase activity of 201.35 nkat/mL followed by CRRIT-2 195.89 nkat/mL. Among four isolates, CRRIT-15 showed 12.60 IU/mL of laccase activity followed by CRRIT-3 9.85 IU/mL of activity (Table 2).

Earlier Druzhinina et al. (2011) reported that *T. reesei* is a major source of hydrolytic enzymes like cellulase and hemicellulase. *T. harzianum* CEN 139, *T.* sp. 104 NH, and *T. harzianum* CEN 155 exhibited 1.74, 1.72, and 1.61 EI value and 0.27, 0.23, and 0.22 IU/mL of endoglucanase activity, respectively (Florencio et al., 2012). Lee et al. (2011) reported *T. harzianum* isolated from post-harvest rice straw possesses 0.095 IU/mL of endoglucanase activity and 0.222 IU/mL of total cellulase activity. Similarly, Pathak et al. (2014) reported *T. harzianum* isolated from soil, rotting wood, and manure from different locales of northern India possesses 1.28 IU/mL endoglucanase and 0.37 IU/mL of total cellulase activity. So, both the isolates NRRIT-26 and NRRIT-27 seem to be a very good potential cellulase and xylanase producer and they may be used as a better option for the preparation of rice straw compost as compared to the previous reports. According to Pathak et al. (2014) 100.2 IU/mL of xylanase activity was observed in the case of *T. harzianum* collected from various location of northern India. NRRIT-26, i.e., *T. reesei* showed highest xylanase activity as compared to the other three isolates that can be considered as the best candidate for rice straw compost. The other three strains, i.e., NRRIT-27, CRRIT-13, and CRRIT-5 also showed higher xylanase activity in comparison to isolates reported by Lee et al. (2011) and Pathak et al. (2014).

Moreover, *T. harzianum* and *T. longibrachiatum* are the wellsprings of laccase production as portrayed by Holker et al. (2002) and Velazquez-Cedeno et al. (2004), respectively. We reported here the creation of laccase enzyme in all the *Trichoderma* isolates isolated from tree bark. *Trichoderma* are adapted well to rice straw and they could be utilized to degrade straw as reported by previous researchers (Kang et al., 2004). Overall, the isolate, CRRIT-15 released the highest number of enzymes as compared to other isolates. Hence, it may be used as a candidate for the preparation of rice straw compost in an economically way.

The *Trichoderma* inoculated straw was decomposed at a faster rate as compared to the non-inoculated one. Among the seven *Trichoderma* strains, NRRIT-15, CRRIT-2, and NRRIT-27 could be able to produce compost from rice straw at a faster rate. There was 18.60, 17.46, and 16.81% of weight loss of the rice straw with NRRIT-15, CRRIT-2, and NRRIT-27 treatments, respectively, after 30 days (Table 3). The loss of weight of the rice straw was 20.95, 18.27, and 18.57%, respectively, after 60 days when treated with NRRIT-15, CRRIT-2, and NRRIT-27. The above mentioned three isolates had also secreted higher quantities of ligno-cellulolytic enzymes as stated above. These enzymes decomposed the rice straw at a faster rate. There were insignificant changes in weight loss after 60 days of incubation as compared to 30 days of incubation. Similarly, the C/N ratio did not vary between 30 and 60 days. So based on these data, we can conclude the compost is generally stable after 30 days of incubation. As *Trichoderma* mediated rice straw compost has a low C/N ratio in *in vitro* condition, the technique can be extended to field conditions which will improve organic matter along with fertility of the soil.

**TABLE 3:** Characteristics of *Trichoderma* mediated-rice straw-compost.

Treatment name	Gravimetric weight loss of decomposed rice straw after 30 days (in percentage)	Gravimetric weight loss of decomposed rice straw after 60 days (in percentage)	C/N (in percentage)
Control	4.48^G^	12.52^E^	67.40^A^
CRRIT-1	7.97^F^	10.49^F^	35.33^B^
CRRIT-2	17.46^B^	18.27^B^	18.27^B^
CRRIT-3	10.76^E^	12.53^E^	34.01^B^
CRRIT-15	18.60^A^	20.95^A^	24.78^F^
CRRIT-16	14.61^C^	15.35^C^	29.72^CD^
CRRIT-22	12.65^D^	13.55^D^	30.39^C^
CRRIT-27	16.81^B^	18.57^B^	28.54^DE^
CV (%)	2.34	1.27	1.38
Tukey’s HSD at 5%	0.8724	0.5605	1.3851

*Means with same letter are not significantly different at p ≤ 0.05.*

### *Trichoderma* as a Plant Growth Promoter

Auxins play a critical role for both the plant growth and root development. The quantity of IAA synthesized by various *Trichoderma* strains in the broth was ranged from 19.19 to 46.28 μg/mL (Table 4). The highest IAA was produced by CRRI-T15 (46.28 μg/mL) which was significantly higher followed by CRRI-T2 (42.38 μg/mL).

**TABLE 4:** Effect of *Trichoderma* application on seedling vigor index of different rice varieties.

Treatment name	Seedling length (cm)		Seedling dry weight (g)		Vigor index-1		Vigor index-2	
	Annapurna	Satabdi	Annapurna	Satabdi	Annapurna	Satabdi	Annapurna	Satabdi
Control	20.10^C^	21.67^D^	0.09^D^	0.11^F^	2210.00^D^	2166.67^D^	9.67^D^	11.00^D^
CRRIT1	24.70^B^	29.03^BC^	0.11^C^	0.14^ E^	2470.00^C^	3036.67^*ABC*^	11.33^C^	14.00^C^
CRRIT2	27.40^AB^	33.03^AB^	0.14^A^	0.19^B^	2740.00^AB^	3336.67^AB^	14.00^A^	17.33^AB^
CRRIT3	26.53^AB^	31.30^*ABC*^	0.14^AB^	0.16^CD^	2653.33^BC^	2996.67^BC^	13.67^AB^	16.00^BC^
CRRIT15	28.57^A^	35.47^A^	0.15^A^	0.21^A^	2923.33^A^	3413.33^A^	14.00^A^	18.33^A^
CRRIT16	26.03^AB^	33.53^AB^	0.12^BC^	0.18^BC^	2603.33^BC^	3153.33^*ABC*^	12.33^BC^	16.00^BC^
CRRIT22	25.03^B^	28.23^C^	0.12^C^	0.16^DE^	2530.00^BC^	2823.33^C^	12.00^C^	15.33^BC^
CRRIT27	24.97^B^	28.03^C^	0.12^C^	0.16^DE^	2436.67^BC^	3257.67^AB^	11.67^C^	15.67^BC^
CV (%)	3.92	5.33	4.27	4.96	3.39	4.46	3.87	5.21
Tukey’s HSD at 5%	2.8722	4.6085	0.0152	0.0232	250.76	388.66	1.3881	2.3207

*Means with same letter are not significantly different at p ≤ 0.05.*

Quantitative assessment of soluble phosphate concentrations in Pikovskaya’s broth was varied from 297.49 to 435.42 μg/mL (Table 2). CRRI-T15 may be treated as the best inducer of phosphate mobilization as it exhibited higher phosphate solubilization capacity in Pikovaskaya’s broth. The amount of inorganic phosphate solubilized was 435.42 μg/mL. Other *Trichoderma* strains, i.e., CRRI-T1, CRRI-T2, CRRI-T3, CRRI-T16, CRRI-T22, and CRRI-T27 were also good phosphate solubilizers.

**FIGURE 3:**
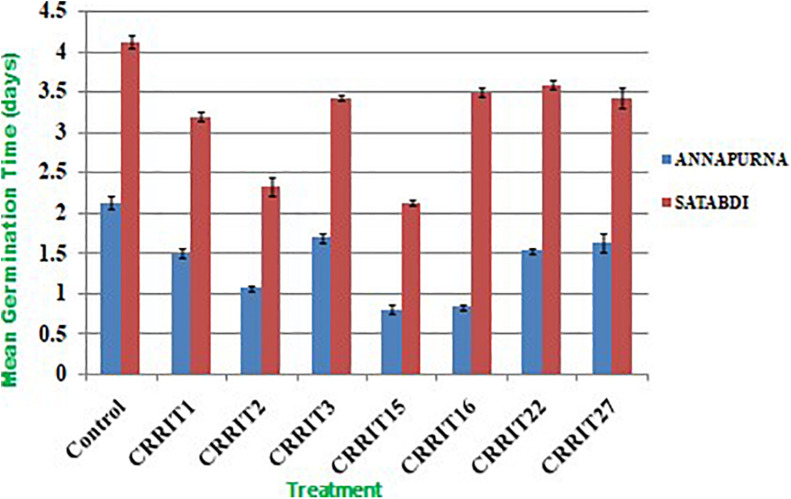
Mean germination time in rice varieties treated with *Trichoderma* and control condition.

Parameters related to seed vigor and seed germination of different *Trichoderma* dressed seeds varied from 2.13–4.13 days (Mean germination time), 2166.67–3413.33 (Vigor index-I) and 11.00–18.33 (Vigor index-II), respectively in variety Satabdi (Figure 3). A similar trend was also found in the case of the Annapurna rice variety. Physiological parameters among the treatments were significantly varied in both the varieties. Absolute chlorophyll content went from 4.61 to 18.78 mg/g in the Annapurna rice variety. Seeds inoculated with *T. hebeiensis* (CRRIT-15), *T. pararees*ei (CRRIT-16), *T. erinaceum* (CRRIT-2), and *T. longibrachiatum* (CRRIT-22) had significantly higher chlorophyll parameters when contrasted with other isolates depicted in the current examination (Table 5). Seed biopriming with beneficial microbes have been reported by several workers for their ability to mitigate biotic stress in an efficient way (Singh P. et al., 2020). During biopriming, antagonistic PGPR increases on the seed surface, thereby defending the plant from pathogen attack and enabling it to be sustained under various stress conditions (Rajput et al., 2019).

**TABLE 5:** Chlorophyll content in different rice varieties due to the application of different *Trichoderma* isolates.

Treatment name	Chl*a* (mg/g of fresh leaf)		Chl*b* (mg/g of fresh leaf)		Chl*a*/Chl*b* (mg/g of fresh leaf)		Total chlorophyll (mg/g of fresh leaf)	
	Annapurna	Satabdi	Annapurna	Satabdi	Annapurna	Satabdi	Annapurna	Satabdi
Control	3.73^E^	1.18^F^	0.87^D^	0.11^F^	4.28^A^	10.78^A^	4.61^E^	1.29^F^
CRRIT1	8.70^D^	8.43^E^	2.38^C^	3.04^E^	3.66^B^	2.77^D^	11.09^D^	11.47^E^
CRRIT2	14.43^A^	15.60^C^	4.35^AB^	4.38^CD^	3.33^*EC*^	3.57^B^	18.78^A^	19.98^C^
CRRIT3	12.71^C^	14.63^D^	4.04^AB^	4.36^D^	3.14^C^	3.36^B^	16.76^C^	18.99^D^
CRRIT15	13.37^BC^	20.55^A^	4.52^A^	6.98^A^	2.98^C^	2.95^CD^	17.89^AB^	27.54^A^
CRRIT16	14.35^A^	15.35^CD^	4.34^AB^	4.84^C^	3.31^*EC*^	3.17^*BCD*^	18.69^A^	20.19^C^
CRRIT22	13.36^BC^	19.51^B^	3.97^B^	5.60^B^	3.36^*EC*^	3.49^B^	17.34^BC^	25.11^B^
CRRIT27	13.87^AB^	15.43^C^	4.06^AB^	4.71^CD^	3.42^*EC*^	3.28^BC^	17.93^AB^	20.14^C^
CV (%)	2.22	1.90	5.06	3.86	4.79	3.33	2.08	1.78
Tukey’s HSD at 5%	0.7542	0.7586	05197	0.4724	0.4736	0.3999	0.9223	0.9277

*Means with same letter are not significantly different at p ≤ 0.05.*

In the field study, all the strains of *Trichoderma* controlled the plant growth along with various agronomical parameters. The highest yield (31.14 g/hill) was recorded from CRRIT-15 followed by CRRIT-16 and CRRIT-13. Similar trends were observed in the case of the Satabdi rice variety (Table 6). As an overall study, all the isolates performed better than the control one (Supplementary Figures 3, 4). Previously Swain et al. (2018) reported higher total chlorophyll content, plant vigor in direct seeded rice treated with *Trichoderma*. Previously Mukherjee et al. (2013) and Mukherjee et al. (2018) explained the role of *Trichoderma* as a plant growth promoter. The enhancement of seed vigor parameters may be due to the production of phenolic compounds and secondary metabolite namely harzianolide by *Trichoderma* spp. (Cai et al., 2013). This result was also found in case of chickpea and wheat (Zhang et al., 2019). All these positive impacts of vigor property helped the plant in uptake and mobilization of nutrients for a longer time, which leads to a better yield.

**TABLE 6:** Growth promotion in different rice varieties due to *Trichoderma* application as indicated by various agronomical parameters.

Treatment name	Dry root weight (g)		Dry shoot weight (g)		Fresh root weight (g)		Fresh shoot weight (g)		Root length (cm)		Shoot length (cm)		Number of tiller/hill		Yield/hill (g)	
								
	Annapurna	Satabdi	Annapurna	Satabdi	Annapurna	Satabdi	Annapurna	Satabdi	Annapurna	Satabdi	Annapurna	Satabdi	Annapurna	Satabdi	Annapurna	Satabdi
Control	0.13^G^	0.20^E^	0.28^C^	0.57^D^	0.32^E^	0.52^F^	1.35^F^	1.30^E^	2.60^D^	1.80^F^	9.54^E^	8.33^D^	12.00^D^	11.00^E^	19.59^F^	22.20^D^
CRRIT1	0.24^F^	0.49^DE^	0.62^C^	1.14^D^	0.46^E^	1.40^E^	1.94^E^	2.62^D^	3.13^D^	2.67^E^	11.41^E^	9.30^D^	14.33^CD^	18.33^CD^	23.46^DE^	33.60^C^
CRRIT2	0.35^E^	1.16^AB^	2.63^B^	2.66^*ABC*^	1.67^B^	2.42^BC^	7.67^B^	6.43^A^	6.57^B^	5.47^BC^	17.37^AB^	17.60^A^	16.67^BC^	23.67^AB^	26.96^BC^	41.38^AB^
CRRIT3	0.79^CD^	0.79^CD^	2.64^B^	2.33^BC^	1.20^D^	1.93^D^	6.77^CD^	5.86^B^	6.13^*B C*^	5.20^C^	16.32^*BCD*^	16.33^B^	18.00^AB^	19.67^C^	29.23^AB^	39.46^B^
CRRIT15	0.96^A^	1.34^A^	3.79^A^	3.05^A^	1.91^A^	2.98^A^	8.72^A^	6.80^A^	7.33^A^	6.17^A^	19.00^A^	18.37^A^	20.33^A^	24.67^A^	31.14^A^	43.53^A^
CRRIT16	0.73^B^	1.00^*ABC*^	2.32^B^	2.95^AB^	1.65^B^	2.23^C^	7.45^BC^	6.30^AB^	6.63^B^	5.70^B^	17.32^*ABC*^	16.00^B^	20.00^A^	21.00^BC^	30.54^A^	40.80^B^
CRRIT22	0.67^C^	0.91^BC^	2.48^B^	2.33^C^	1.38^C^	1.77^D^	6.32^DE^	3.37^C^	5.80^C^	4.47^D^	14.45^D^	14.67^C^	15.00^C^	16.00^D^	22.73^E^	36.15^C^
CRRIT27	0.62^D^	1.01^*ABC*^	2.27^B^	2.53^*ABC*^	1.22^CD^	2.61^B^	5.91^E^	6.66^A^	6.73^AB^	5.20^C^	15.33^CD^	16.10^B^	19.33^A^	20.67^C^	25.60^CD^	40.38^B^
CV (%)	2.59	13.82	9.42	9.79	5.08	5.08	4.33	3.59	3.76	3.28	4.57	2.84	5.36	4.79	3.17	2.45
Tukey’s HSD at 5%	0.0402	0.3431	0.5777	0.6191	0.1794	0.2905	07198	0.5089	0.6083	0.4327	1.9868	1.2063	2.6206	2.6766	2.391	2.629

*Means with same letter are not significantly different at p ≤ 0.05.*

Among the secondary metabolites, IAA (auxin) helps in plant growth and potentially increases the root length as well. Laboratory studies have emphasized the role of plant growth promoting fungi as auxin producers and biocontrol operators (Hossain et al., 2007; Contreras-Cornejo et al., 2009) in plant development. This key hormone was synthesized by the fungus *Trichoderma* in symbiotic as well as in pathogenic interactions (Gravel et al., 2007). Similarly, plants can only uptake and mobilize essential micronutrients if they are solubilized by microbes (Rudresh et al., 2005). As indicated by Dunaitsev et al. (2008) *Trichoderma* spp. can deliver phosphate from mineral crude materials as plant accessible structures. It was seen that *Trichoderma* isolates a demonstrated higher capacity to solubilize the phosphate as they additionally displayed great reactions to plant growth promotion action after direct seed treatments. *Trichoderma* as plant symbionts for updated supplement take-up, extended root and shoot advancement, improved plant influence and biotic/abiotic stress flexibility have been widely discussed (Harman, 2011).

### Improvement of Plant Resistance by the Articulation of Stress Responsive Enzymes

Inside and out higher verbalization of the enzymes related to stress was seen in *Trichoderma* seed treated plants when appeared differently in relation to untreated plants against both the rice assortments. Correspondingly, CRRIT-15, and CRRIT-2 treated root and shoots of rice assortment Annapurna and Satabdi had exceptionally higher PER, SOD, PPO, and TP activity contrasted with other treatment. Also, catalase action was higher in CRRIT-15 and CRRIT-2 treatment in both root and shoot. Comparative examples were found in peroxidase action in root and shoot of both the rice assortments (Table 7).

**TABLE 7:** Expression of defense enzymes related to stress in the rice varieties.

Treatment name	Expression of catalase (in unit/min/gm)				Expression of peroxidase (in unit/min/gm)				Expression of superoxide dismutase (in unit/min/gm)				Expression of polyphenol oxidase (in unit/min/gm)				Expression of total phenolics (in unit/min/gm)			
					
	Annapurna		Satabdi		Annapurna		Satabdi		Annapurna		Satabdi		Annapurna		Satabdi		Annapurna		Satabdi	
										
	ROOT	SHOOT	ROOT	SHOOT	ROOT	SHOOT	ROOT	SHOOT	ROOT	SHOOT	ROOT	SHOOT	ROOT	SHOOT	ROOT	SHOOT	ROOT	SHOOT	ROOT	SHOOT
Control	5.00^E^	7.00^E^	5.00^F^	5.67^E^	0.51^F^	0.39^E^	0.52^D^	0.42^*H*^	3.56^E^	5.98^E^	4.39^G^	3.63^G^	20.49^D^	24.99^D^	19.23^D^	27.55^D^	0.11^G^	3.48^G^	0.10^G^	3.73^G^
CRRIT1	9.00^D^	18.33^D^	10.67^E^	10.33^D^	0.72^E^	0.84^D^	0.66^D^	0.67^G^	7.78^CD^	8.50^D^	5.57^F^	5.43^F^	25.85^C^	30.35^C^	24.60^C^	32.92^C^	0.87^F^	4.37^F^	0.86^F^	4.62^F^
CRRIT2	20.67^A^	27.67^AB^	20.33^B^	24.67^AB^	1.28^AB^	1.89^A^	2.11^A^	1.49^C^	10.58^A^	12.42^A^	10.31^B^	11.64^B^	58.78^A^	63.28^A^	57.53^A^	65.85^A^	4.20^B^	7.70^B^	4.19^B^	7.95^B^
CRRIT3	18.33^B^	26.00^BC^	19.67^BC^	23.33^B^	1.07^C^	1.46^B^	1.66^C^	1.13^E^	9.59^B^	10.27^C^	9.76^C^	10.44^C^	41.15^B^	45.65^B^	39.90^B^	48.21^B^	2.90^D^	6.40^D^	2.89^D^	6.66^D^
CRRIT15	21.67^A^	28.67^A^	22.33^A^	26.33^A^	1.33^A^	1.98^A^	2.18^A^	1.92^A^	11.14^A^	12.82^A^	12.62^A^	12.51^A^	60.02^A^	64.52^A^	58.76^A^	67.08^A^	4.73^A^	8.23^A^	4.72^A^	8.49^A^
CRRIT16	18.33^B^	24.33^C^	18.67^CD^	23.33^B^	1.14^BC^	1.03^C^	1.82^B^	1.25^D^	8.12^C^	10.45^C^	8.65^D^	9.69^D^	41.95^B^	46.45^B^	40.70^B^	49.01^B^	3.04^D^	6.54^CD^	3.03^D^	6.79^CD^
CRRIT22	16.33^C^	20.67^D^	17.67^D^	19.67^C^	0.86^DE^	1.10^C^	1.55^C^	0.98^F^	7.19^D^	9.99^C^	7.55^E^	8.51^E^	27.97^C^	32.47^C^	26.72^C^	35.04^C^	2.01^E^	5.51^E^	1.99^E^	5.76^E^
CRRIT27	18.67^B^	26.33^*ABC*^	19.33^BC^	25.33^A^	1.02^CD^	1.61^B^	1.87^B^	1.62^B^	9.73^B^	11.45^B^	9.89^ BC^	10.44^C^	43.47^B^	47.97^B^	42.21^B^	50.53^B^	3.43^C^	6.93^C^	3.42^C^	7.19^C^
CV (%)	3.23	3.73	3.11	3.09	6.03	4.64	3.30	3.17	3.18	3.14	2.08	2.01	2.47	2.22	2.55	2.10	4.63	2.29	4.65	2.19
Tukey’s HSD at 5%	1.4911	2.4043	1.4952	1.7643	0.1721	0.1721	0.1468	0.1081	0.7743	0.9249	0.5144	0.5233	2.8383	2.8383	2.8383	2.8383	0.3549	0.4046	0.3549	0.4046

*Means with same letter are not significantly different at p ≤ 0.05.*

In blend with their immediate impact on the pathogen structure and action, *Trichoderma* spp. has additionally been found to invigorate plant resistance mechanisms (Yedidia et al., 2001). Successful *Trichoderma* strains can instigate a more grounded reaction in the plant contrasted with pathogen-triggered immunity by creating an assortment of microorganisms related molecular patterns (MAMPs), for example, hydrophobins, expansin-like proteins, metabolites, and catalysts like catalase, peroxidase, and superoxide dismutage, having a direct antimicrobial movement (Vargas et al., 2008). *Trichoderma* can likewise improve ETI by causing a quicker reaction (preparing), or initiate it by discharging exacerbates that, similarly as with some pathogen molecules, are explicitly perceived by plant cell receptors (Bailey et al., 1993). In the present exploration, the stress enzymes expressed in a higher amount in *Trichoderma* treatments rather than the control group. Mohapatra and Mittra (2017) and Swain et al. (2018) documented that *Trichoderma* spp. triggered the fabrication of antioxidant enzymes in wheat and rice seedlings, respectively. *Trichoderma* isolates were accounted to help fundamental defense reactions through different catalysts (Małolepsza et al., 2017). PPO is engaged with the plant guard system against pathogens by catalyzing the oxidation of phenols to quinines in an oxygen-subordinate way (Daw et al., 2008). An increase in defense enzymes activity (Mandal et al., 2013) and TP content (Mandal et al., 2013) in plants with a reaction to pathogen assault has also been previously reported.

### *Trichoderma* Seed Biopriming Induces Antioxidant Gene Expression in Rice Plants

**FIGURE 4:**
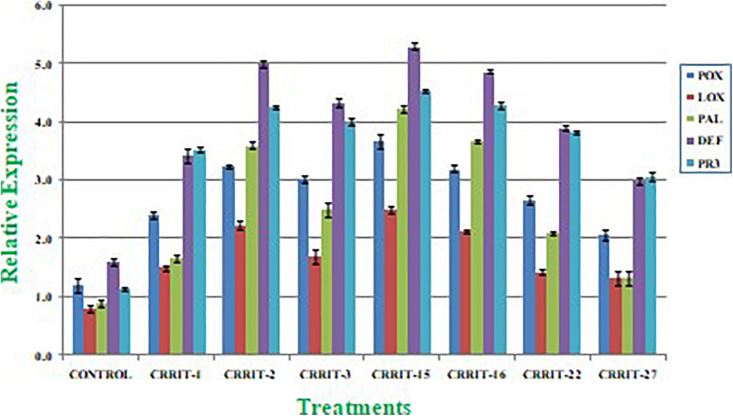
Expression of antioxidant and defense related genes in rice variety (Annapurna). Results are expressed as means of three replicates and vertical bars indicate the standard deviation of the means.

**FIGURE 5:**
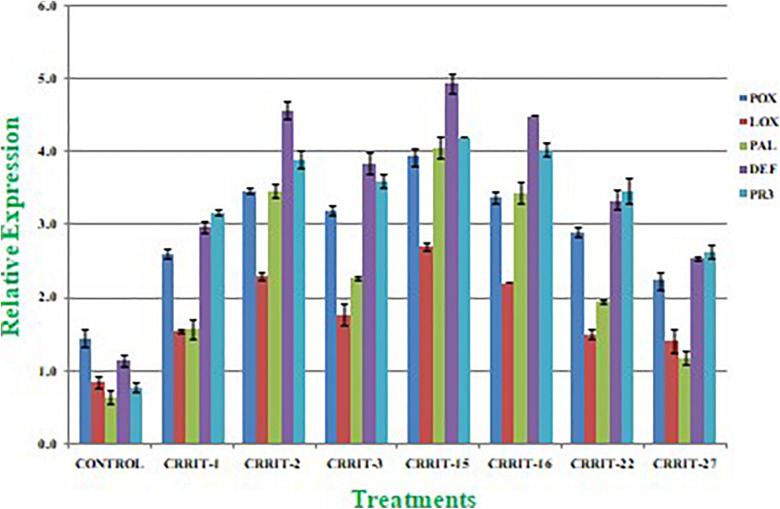
Expression of antioxidant and defense related genes in rice variety (Satabdi). Results are expressed as means of three replicates and vertical bars indicate the standard deviation of the means.

Plants have created solid cell antioxidant molecular systems because of the utilization of biocontrol specialists like *Trichoderma* (Rejeb et al., 2014; Swain et al., 2018), however the initiation and articulation of these genes change under plant organism communication conditions (Singh D. P. et al., 2020). Albeit, both organism-treated and non-treated plants were developed under typical development conditions. We investigated the plant molecular reactions as far as the outflow of prominent defense (PAL and DEFENSIN) and antioxidant (POX, LOX, and PR-3) genes following microbial inoculation (Table 8). We observe multifold over expression of genes in both the strains. However, *Trichoderma* seed biopriming caused >2-fold up regulation of every gene in both the rice varieties. Besides, CRRIT-15 exhibited the highest level of fold expression (i.e., >3) of all the genes as compared to control one. CRRIT-16 and CRRIT-2 treatment performed the second highest level of fold expression (i.e., >2.5) by following CRRIT-15 (Figures 4, 5).

**TABLE 8:** Details of primers used in antioxidant and defense related gene expression study.

Serial No.	Primer name	Gene name	Primer sequence 5′-3′
1	Rice (Actin)F	Actin	CTGCTGGAATGTGCTGAGAGAT
	Rice (Actin)R		CGTCTGCGATAATGGAACTGG
2	Rice (POX)F	Peroxidase	CATGCTACTGCTCACCTTTGA
	Rice (POX)R		TCACTCTAGGTGGGATATACT
3	Rice (LOX)F	Lipoxygenase	AGATGAGGCGCGTGATGAC
	Rice (LOX)R		CATGGAAGTCGAGCATGAACA
4	Rice (PAL)F	Phenylalanine ammonia lyase	GGTGTTCTGCGAGGTGATGA
	Rice (PAL)R		AGGGTGGTGCTTCAGCTTGT
5	Rice (Defensin)F	Defense Enzyme	CCGGCGAACTGCGTGTAC
	Rice (Defensin)R		GGCGTCGAGCAGAATTGG
6	Rice (PR-3)F	PR Protein	TACTGTGTCCAGAGCTCGCAGTGG
	Rice (PR-3)R		TCTGGTTGTAGCAGTCCAAGTTGG

Seed biopriming with beneficial microbes have been accounted for their capacity to relieve biotic stress in an effective way. During the process of seed biopriming, antagonistic plant growth promotive activity increases on the seed surface, hence not only defending the plant from pathogen attack but also promoting the plant growth (Swain et al., 2018; Rajput et al., 2019). Co-vaccination of *Paenibacillus polymyxa* and *Rhizobium tropici* mitigated drought in common bean (*Phaseolus vulgaris* L.) (Figueiredo et al., 2008). In the present study it was found that *Trichoderma* treated plants exhibited an increase in total phenol content catalase content, peroxidase content superoxide dismutase content and expression of defense gene (POX, LOX, PAL, DFENSIN, and PR-3) as compared to control one. This further authenticates the induction of growth responses and defense response in rice up on application of *Trichoderma* in rice seeds. Plant phenolics are normally framed in light of both biotic and abiotic stress through enactment of phenyl propanoid pathway and include in cellulase, lignin, xylanase, and biosynthesis. Thus, PPO catalyzes phenolics, exacerbating the production of quinines through a secondary reaction, which further prompts the arrangement of an earthy colored complex polymer, melanin; a physical hindrance to microbe ingression (Taranto et al., 2017). In this study all the *Trichoderma* strains exhibited higher total phenol content, PPO activity as compared to the control one. Besides, CRRIT-15, CRIT-16, and CRRIT-2 out-perform the others. This suggests a synergistic effect of *Trichoderma* in induction of plant growth and defense in rice varieties. Notwithstanding these, acceptance of different development promotive genes and antioxidant agent responsive genes mitigates oxidative stress in plant cells. Our outcomes are validated with Singh et al. (2013) in which they revealed improved movement of PAL, PPO, PO, and SOD content in chickpea treated with *Pseudomonas*, *Trichoderma*, and *Rhizobium*. Contrasting these discoveries and our outcomes prompts the presumption that stronghold of rice with microbial inoculants characteristically balanced molecular components to give resilience against ROS scavenging in a manner to making plants fortified against stress difficulties. Rice seed biopriming of *Trichoderma* could, accordingly, become a proficient methodology for raising yield for better profitability and resistance against stress conditions.

## Conclusion

In India alone, an excess of 250 commercial formulations are available, however, a large portion of them are from a solitary strain, i.e., *Trichoderma viride* (presently renamed as *Trichoderma asperelloides*). Most of the *Trichoderma* strains defined in literature were isolated from the soil or rhizosphere, but very few are isolated from the above ground aerial parts. In the present study we evaluated both the biocontrol and growth promotion activity of seven different *Trichoderma* strains isolated from above ground parts. The rice seed treatment with *Trichoderma* strains not only promoted germination, seedling vigor, and growth of the plant, but also increase the level of gene expression related to plant defense. Apart from growth promotion these strains imparted intrinsic stress tolerance to rice by producing a higher amount of defense enzymes like catalase, peroxidase, superoxide dismutase, polyphenol oxidase, and total phenolics content as evidenced by the expression of their respective genes. Our recent investigation tries to fill the gap by isolating and identifying above ground *Trichoderma* strains for rice health management. Two strains, namely *T. hebeiensis* and *T. erinaceum* may be promoted in sustainable crop management for their beneficial role.

## Data Availability Statement

The raw data supporting the conclusions of this article will be made available by the authors, without undue reservation.

## Author Contributions

HS: investigation, statistical analysis, and writing – original draft. TA: conceptualization and writing – review and editing. AM: conceptualization, writing – review and editing, project administration, resources, and supervision. PS, SS, AK, and RJ: investigation. PB: methodology. SN: writing – review and editing, and project administration. SM: project administration. MB: statistical analysis. SKM and NZ: conducted the field trials and recorded the data. All authors contributed to the article and approved the submitted version.

## Conflict of Interest

The authors declare that the research was conducted in the absence of any commercial or financial relationships that could be construed as a potential conflict of interest.

## Footnotes

1 http://www.isth.info/tools/blast/markers.php

2 www.iasri.res.in/sscnars/-
