# Supplementary material for: Seed Biopriming With Trichoderma Strains Isolated From Tree Bark Improves Plant Growth, Antioxidative Defense System in Rice and Enhance Straw Degradation Capacity
Source: Front Microbiol. 2021 Feb 26;12:633881. doi: 10.3389/fmicb.2021.633881 (PMC7952651; doi:10.3389/fmicb.2021.633881)
Supplement: Supplementary file 1 [file Presentation_1.PPT]

## Slide 1
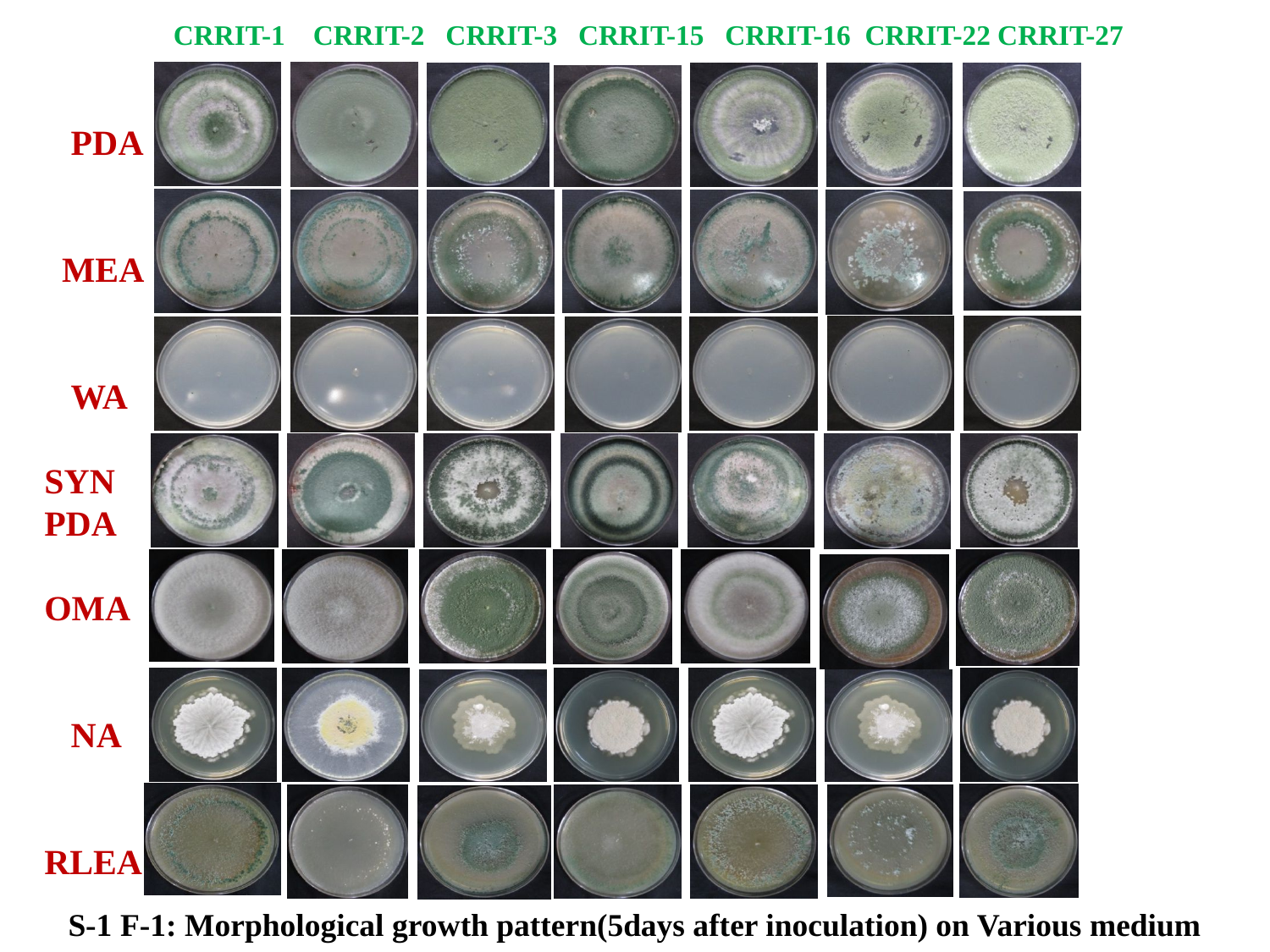

CRRIT-1 CRRIT-2 CRRIT-3 CRRIT-15 CRRIT-16 CRRIT-22 CRRIT-27
 PDA
 MEA
 WA
SYN PDA
OMA
 NA
RLEA
S-1 F-1: Morphological growth pattern(5days after inoculation) on Various medium

## Slide 2
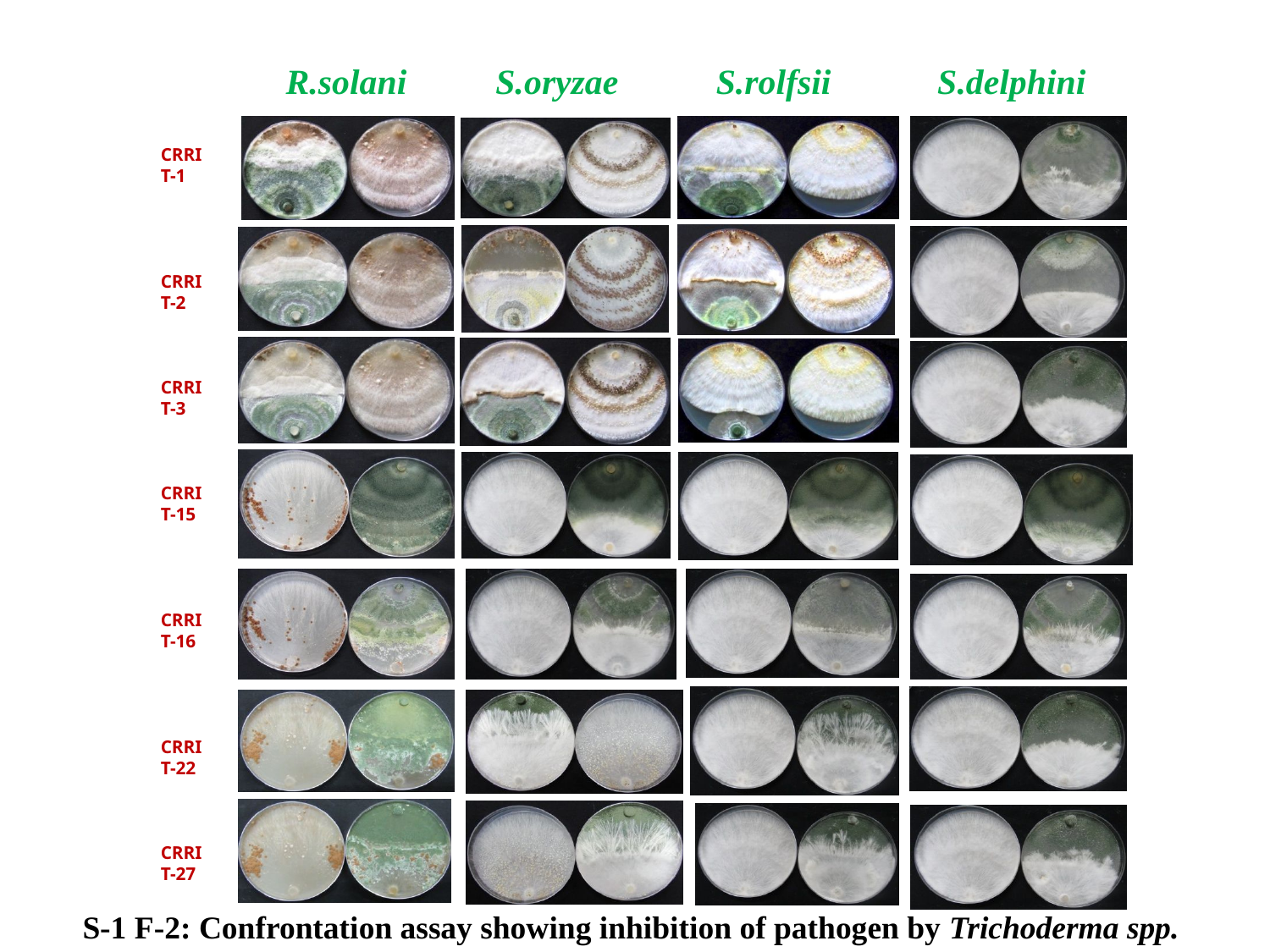

R.solani S.oryzae S.rolfsii S.delphini
CRRIT-1
CRRIT-2
CRRIT-3
CRRIT-15
CRRIT-16
 CRRIT-22
CRRIT-27
S-1 F-2: Confrontation assay showing inhibition of pathogen by Trichoderma spp.

## Slide 3
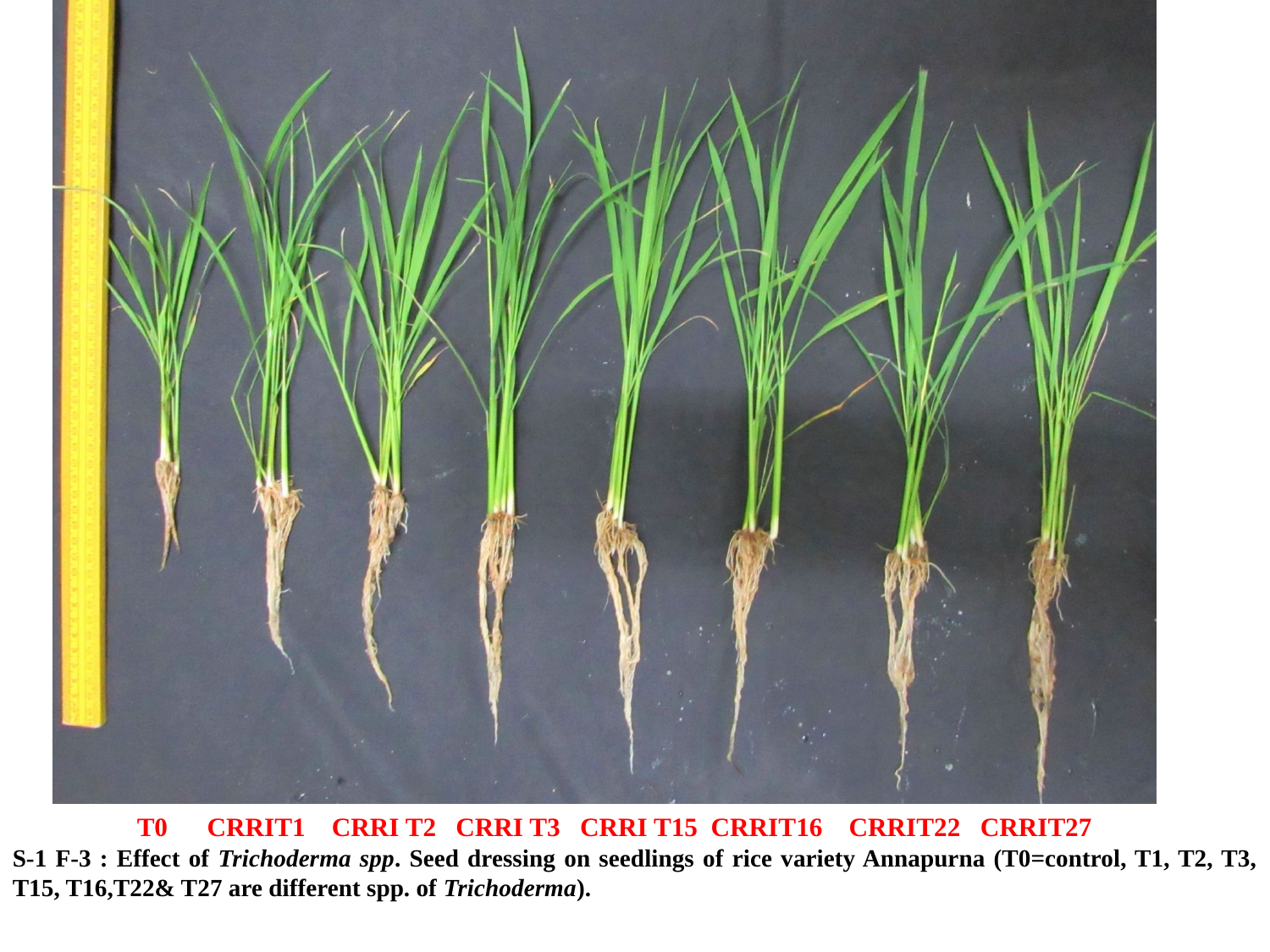

T0 CRRIT1 CRRI T2 CRRI T3 CRRI T15 CRRIT16 CRRIT22 CRRIT27
S-1 F-3 : Effect of Trichoderma spp. Seed dressing on seedlings of rice variety Annapurna (T0=control, T1, T2, T3, T15, T16,T22& T27 are different spp. of Trichoderma).

## Slide 4
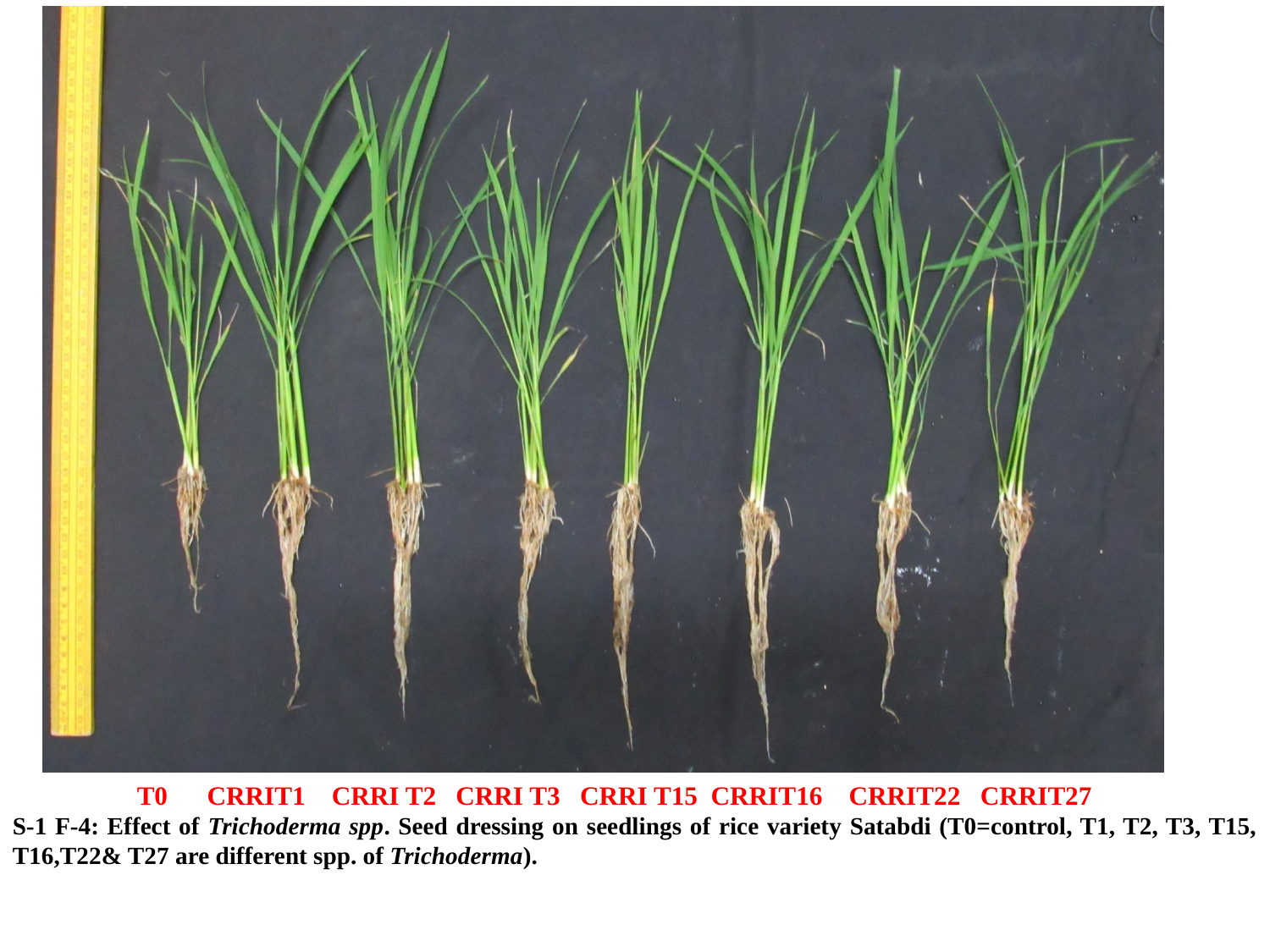

T0 CRRIT1 CRRI T2 CRRI T3 CRRI T15 CRRIT16 CRRIT22 CRRIT27
S-1 F-4: Effect of Trichoderma spp. Seed dressing on seedlings of rice variety Satabdi (T0=control, T1, T2, T3, T15, T16,T22& T27 are different spp. of Trichoderma).
